# Parent Genotype and Environmental Factors Influence Introduction Success of the Critically Endangered Savannas Mint (*Dicerandra immaculata* var. *savannarum*)

**DOI:** 10.1371/journal.pone.0061429

**Published:** 2013-04-09

**Authors:** Cheryl L. Peterson, Gregory S. Kaufmann, Christopher Vandello, Matthew L. Richardson

**Affiliations:** 1 Rare Plant Conservation Program, Bok Tower Gardens, Lake Wales, Florida, United States of America; 2 Florida Department of Environmental Protection, Florida Park Service, Bureau of Natural and Cultural Resources, Tallahassee, Florida, United States of America; 3 Florida Department of Environmental Protection, Florida Park Service, Savannas Preserve State Park, Jensen Beach, Florida, United States of America; 4 USDA-ARS, U.S. Horticultural Research Laboratory, Subtropical Insects Research Unit, Fort Pierce, Florida, United States of America,; Wuhan Botanical Garden, Chinese Academy of Sciences, China

## Abstract

Species previously unknown to science are continually discovered and some of these species already face extinction at the time of their discovery. Conserving new and rare species in these cases becomes a trial-and-error process and conservationists will attempt to manage them by using knowledge of closely related species, or those that fill the same ecological niche, and then adapting the management program as needed. Savannas Mint (*Dicerandra immaculata* Lakela var. *savannarum* Huck) is a perennial plant that was discovered in Florida scrub habitat at two locations in 1995, but is nearly extinct at these locations. We tested whether shade, leaf litter, propagation method, parent genotype, parent collection site, planting date, and absorbent granules influenced survival, reproduction, and recruitment of Savannas Mint in a population of 1,614 plants that we introduced between June 2006 and July 2009 into a state protected site. Survival and reproduction of introduced plants, and recruitment of new plants, was higher in microhabitats in full sun and no leaf litter and lower in partially shaded habitats. The two sites from which parent plants were collected differentially influenced survival and reproduction of introduced plants. These differences in survival and reproduction are likely due to underlying genetic differences. Differential survival of progeny from different parent genotypes further supports the idea that underlying genetics is an important consideration when restoring plant populations. The most successful progeny of parent genotypes had survival rates nearly 12 times higher than the least successful progeny. We speculate that many of these environmental and genetic factors are likely to influence allopatric congeners and other critically endangered gap specialists that grow in Florida scrub and our results can be used to guide their conservation.

## Introduction

Species previously unknown to science are continually discovered and some of these species are already threatened with extinction at the time of their discovery (e.g. [1], [2]). An estimated 60,000 species of plants remain unknown to science and many are endemic only to biodiversity hotspots that have high levels of habitat loss, so they are likely to be rare and in shrinking habitats when discovered [3]–[5]. Protected conservation areas, which are often within biodiversity hoptspots, are a crucial component of management programs for rare species because they can serve as experimental translocation sites [6], [7].

Florida scrub habitat, shrubland on infertile, xeric sandy ridges [8], [9], is a hotspot for endemic and endangered plants [10]–[12]. The plant genus *Dicerandra* (Lamiaceae) contains four annual and five perennial species that are restricted to scrub and sandhill habitats primarily in Florida [13], [14] and is the highest ranked genus of rare southeastern endemic plants [12]. Six of the nine species have extremely small geographic ranges, there is probably little gene flow between populations, and their habitats are being converted for agricultural and urban development [10], [12], [15]–[17]. Some *Dicerandra* spp. are listed on state and federal endangered species lists, but this affords little protection and populations continue to decline (e.g. [18]).

Savannas Mint (*Dicerandra immaculata* Lakela var. *savannarum* Huck) is a perennial plant that was discovered in St. Lucie County, FL in 1995 at two locations separated by less than 0.3 km [19]. Savannas Mint was originally considered a wide-leafed variety of Lakelas Mint (*D. immaculata* var. *immaculata*) based on morphological similarity of the two varieties, and its scientific name still reflects this consideration [19]. However, Savannas Mint has floral volatiles that differ from Lakelas Mint (unpublished data) and a relatively recent phylogenetic analysis shows that Savannas Mint has divergent nuclear and chloroplast genomes that make it unique in the *Dicerandra* genus and necessary to conserve in order to protect the genetic diversity of the genus [14].

Savannas Mint is likely dependent on regular disturbance for survival because it is endemic to Florida scrub habitat. Florida scrub habitat often has a canopy of pine, oak, and hickory that is periodically top-killed by fire, which promotes gaps and maintains a shrubby habitat structure [9]. Canopy openings created by fire allow populations of endangered plants endemic to Florida scrub, including other *Dicerandra* spp., to increase [18], [20]–[24]. Survival and reproduction of gap specialists of Florida scrub decline with increasing time post-disturbance [18], [23], [25], [26]. Some *Dicerandra* spp. are known to be killed by fire, but their populations persist because some mature plants that were untouched by fire survive to reproduce or new individuals recruit from seeds in the soil [18].

**Figure 1:**
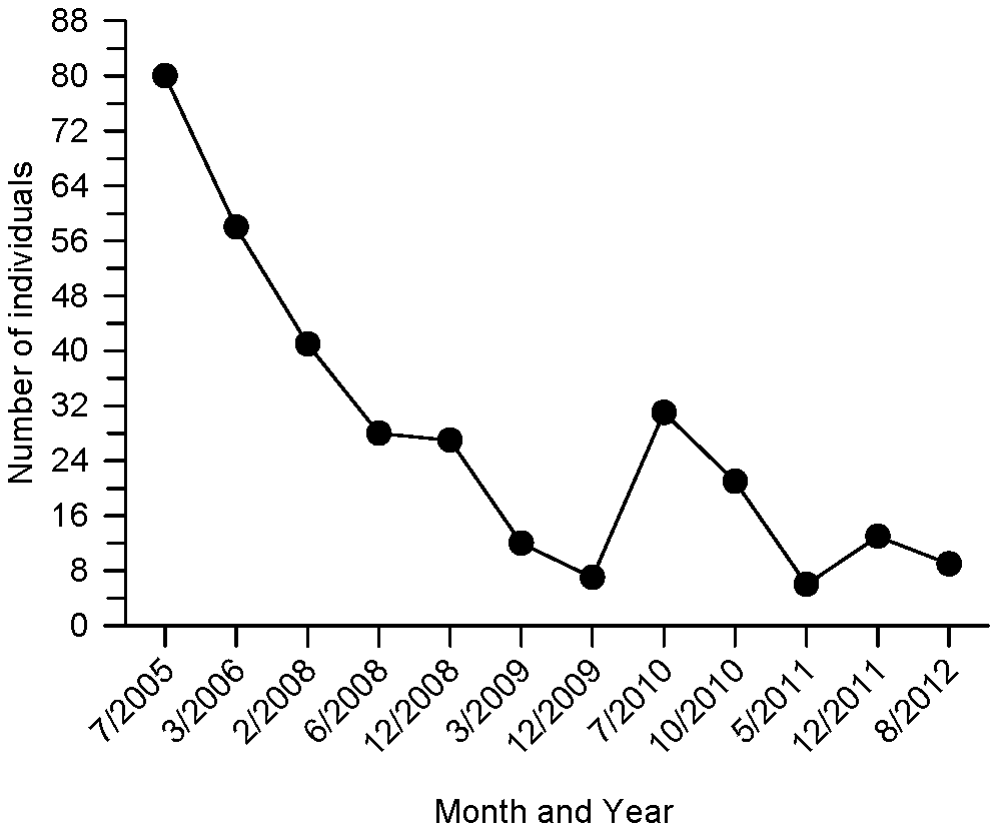
Number of individual plants in the last known wild population of *Dicerandra immaculata* var. *savannarum.*

The two sites where Savannas Mint were discovered were not protected or managed for this gap specialist: one site was located within a brush and tree line between the Florida East Coast Railway (FECR) and Savannas Preserve State Park (SPSP) and the second was located along a residential road (Eden Creek Lane; EC) that spanned three private properties within a subdivision. These are the only locations known to support Savannas Mint (unpublished data) and likely supported a single contiguous population before construction of the railroad and other anthropogenic disturbances. Approximately 200 individuals were counted on well-drained ridge habitat at these locations in 1995 [19] and this declined to 89 by 2005 (unpublished data). The survival of Savannas Mint at FECR was threatened by discarded debris and overgrowth of native and invasive plant species, so all individuals were removed and transported to Bok Tower Gardens (Lake Wales, FL) in 2005. Savannas Mints at EC have been regularly counted since 2005 and the population has steadily declined nearly to extinction (Fig. 1). The mortality rate at EC is high during years of drought and when the site is not hand-cleared of taller vegetation that blocks sunlight. No new plants have been recruited to the population recently (unpublished data).

We have been involved in conservation efforts aimed at preserving germplasm from all 89 plants surveyed during 2005, propagating new individuals, and establishing and monitoring a new population of Savannas Mint within SPSP, which is adjacent to EC and FECR [27]. However, nothing has been published in the primary literature about this species, except the original discovery and a phylogeny [14], [19]. Therefore, to guide the establishment of this new population at SPSP, we relied on what little information was available about the ecological niches of related *Dicerandra* spp. [24], lessons learned from our restoration of Lakelas Mint [28], and observations of habitat that has become unsuitable at EC. Environmental and genetic factors individually and interactively influence survival and reproduction of plants (e.g., [29]–[31]), so we tested the influence of shade, leaf litter, propagation method, parent genotype, parent collection site, planting date, and absorbent granules on survival, reproduction, and recruitment of Savannas Mint in the introduced population, and we present those findings here.

## Methods

SPSP encompasses more than 2,670 ha and contains fire-dependent natural communities, such as pine flatwoods, wet prairie, basin marsh, and scrub. The scrub habitats within SPSP are part of the larger Atlantic Coastal scrub ridge, a relic dune system in which many endemic species persist. The area where we introduced Savannas Mint within SPSP is located approximately 5 km north of the wild population at EC and contains coastal scrub on well-drained sand, which is similar to the historical habitat. Seeds were collected annually in December from 2005–2009 and cuttings were made for clonal propagation approximately six months prior to each date we planted new individuals at SPSP. Cuttings rooted within 2–6 weeks and up to 90% survived. We followed the Center for Plant Conservation's guidelines when collecting seeds and cuttings to prevent a negative impact on the dynamics of the wild population [32].

On seven dates between June 2006 and July 2009, 1,614 young plants were introduced at SPSP. Plants were watered three times per week for the first month after planting and at least two times per week for the next two months unless rainfall measured at least 2.5 cm during a week. On the first four planting dates (June, September, and November 2006 and February 2007), 632 plants were placed primarily within four microhabitats: full sun and no leaf litter (n = 125), full sun and leaf litter (n = 144), partial shade and no leaf litter (n = 207), and partial shade and leaf litter (n = 156). Shade and leaf litter was produced primarily by sand pine [*Pinus clausa* (Chapm. Ex Engelm.) Vasey ex Sarg.], but also species of oak (*Quercus* spp.) and scrub hickory (*Carya floridana* Sarg.). The remaining 982 plants were placed primarily in partial shade and leaf litter on the final three dates (October 2007, September 2008, and July 2009). Across these three final dates, a product called Zeba® Quench™ (Absorbent Technologies, Inc., Beaverton, OR), which consists of starch-based superabsorbent granules, was added to the soil below some of the plants because it supposedly absorbs water and releases it to the roots of the plants. We predicted that Quench™ would increase survival, especially during times of high heat and low rainfall when the weekly watering regime may have been insufficient and after the watering regime was discontinued.

The site from which the parent plant was collected (i.e., EC or FECR) and the propagation method (i.e., seed or clonal propagation) was noted for each of the 1,614 plants. Additionally, we noted the parent plant for each plant that was clonally propagated, and we considered each parent a separate genotype (following the method of [33]). Plants were individually tagged and the survival and reproductive stage (hereafter “reproduction”) of each one was noted in the field during November or December yearly through 2011. A plant was considered reproductive if it was flowering or producing fruits (including empty calyxes) on the observation date. We tagged new plants recruited to the population in December 2011 and established two plots (circular plots each with a radius of approximately 5.6 meters) in each of the four microhabitat types (N = 8) to compare recruits among these microhabitats.

### Statistical Analyses

The influence of microhabitat, parent collection site, propagation method, and planting date on survival and reproduction of the 632 plants introduced during the first four planting dates was determined with binomial regression models (PROC GENMOD, [34]). Our primary interest with this analysis was to determine how microhabitat, and interactions with parent collection site, propagation method, and planting date, influenced short (1 yr) and long-term (4 yr) survival and reproduction. Therefore, we also included those interactions in our models, but removed them if they were not significant [35]. We used survival and reproduction data collected one and four years after each plant's planting date in our analysis. The LSMEANS statement was then used to estimate separation between pairs of means on a log scale [34], [35]. We tested for differences in the number of newly recruited plants among types of microhabitat with a Poisson regression model (PROC GENMOD, link  =  log option, [34]), with the percent of introduced plants (i.e., parent plants of the recruits) surviving to one year post-planting as a covariate. The Poisson regression model was corrected for over-dispersion (dscale option, proc GENMOD) and we used the LSMEANS statement to separate pairs of means on a log scale [34], [35].

We also used binomial regression models with means separation to investigate the influence of 1) Quench™ on the two-year survival and reproduction of 1,046 and 371 plants, respectively, planted between October 2007 to July 2009, 2) parent collection site, propagation method, and planting date on the two year-survival and reproduction of 1,503 and 525 plants, respectively, across all seven planting dates, and 3) 50 parent genotypes on the two-year survival of 1,116 clonal progeny, planted across all seven planting dates. Our sample sizes differ among these three analyses, and from the analysis in the prior paragraph, because treatments were not applied to all plants, such as microhabitat and Quench™, or were applicable only for clonal plants, such as the influence of parent genotype. We chose these 50 parent genotypes because each genotype had a minimum of ten progeny. The influence of parent genotype on reproduction was not analyzed because too few individual plants survived to reproduce. In the prior paragraph we analyzed survival and reproduction data collected one and four years after the planting date of each plant because we were interested in immediate and long-term effects. However, for the analyses in this paragraph we analyzed survival and reproduction two years after the planting date of each plant because this approximates the average life span for introduced plants of this species (unpublished data). Therefore, any differences in survival and reproduction due to environmental or genetic factors should be evident within this time.

### Ethics Statement

All seeds and cuttings collected for propagation in this study were performed with permission from private landowners and with the required listed species harvest permits from the state of Florida, Department of Agriculture and Consumer Services, Division of Plant Industry. SPSP is protected, managed, and publicly owned. No seeds or cuttings were collected from the introduced population at SPSP and all research at this location was approved by the Florida Department of Environmental Protection, Division of Recreation and Parks, District 5 Administration.

## Results

### Influence of microhabitat on survival, reproduction, and recruitment of Savannas Mint

**Figure 2:**
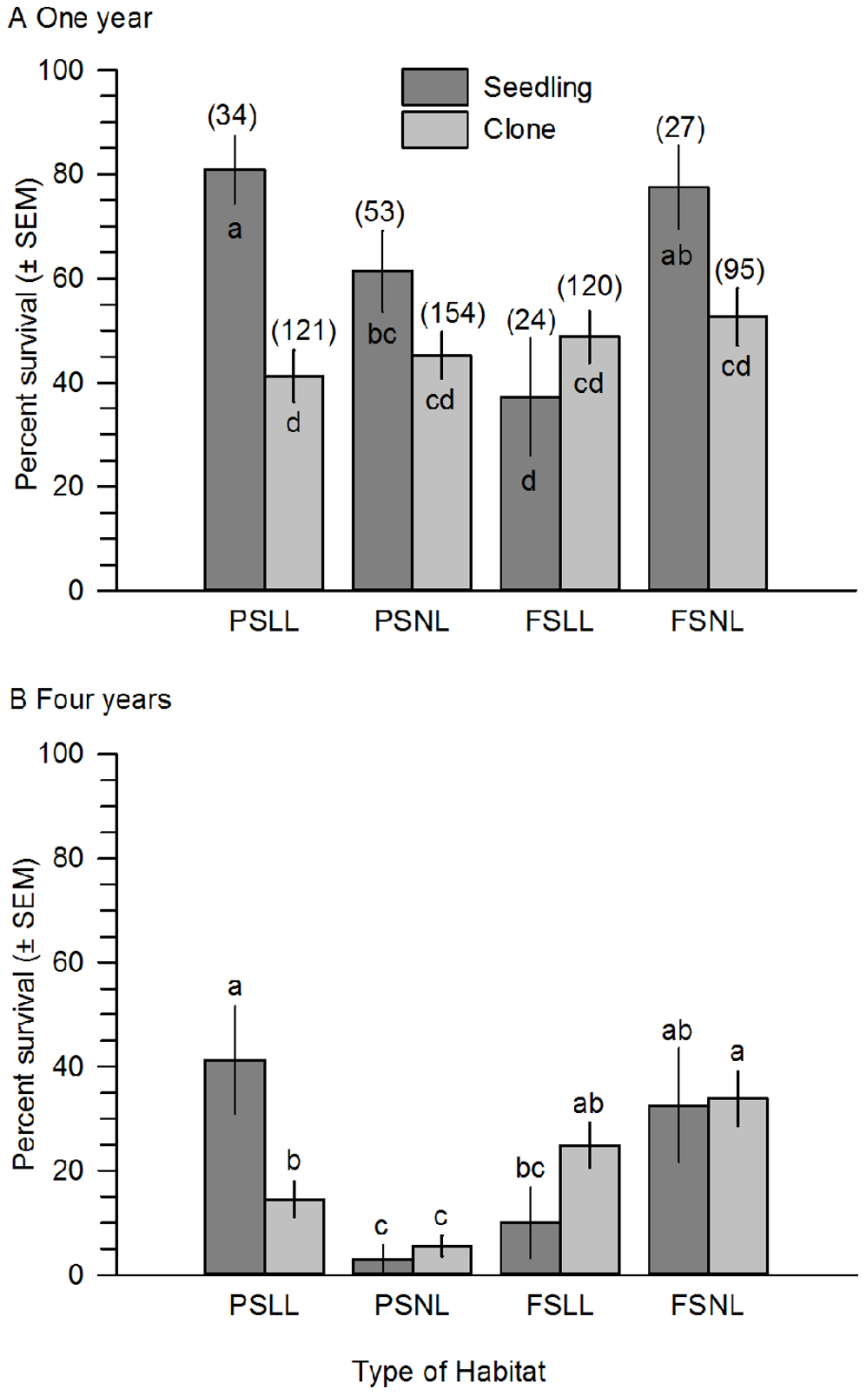
Mean percent (± SEM) survival of Dicerandra immaculata var. savannarum at Savannas Preserve State Park, FL. A. One year post-planting. B. Four years post-planting. Plants were propagated from seeds or clonal cuttings and planted in four types of microhabitat. Microhabitat types: PSLL  =  part shade and no leaf litter; PSNL  =  part shade and no leaf litter; FSLL  =  full sun and leaf litter; and FSNL  =  full sun and no leaf litter. Sample sizes in parentheses for each microhabitat type. Means with different letters are significantly different (means separation test, P<0.05).

Survival of Savannas Mint one and four years post-planting was influenced by the interaction of microhabitat type with propagation method, and the collection site of the parent and planting date also were significant (Table 1). Survival of plants was higher, on average, one year post-planting when propagated from seeds and planted either in part shade and leaf litter or full sun and no leaf litter (Fig. 2a). The survival of clonally propagated plants was not differentially influenced by microhabitat and was low compared to plants propagated from seed (Fig. 2a). Survival was similarly influenced four years-post planting, except that plants propagated from clonal cuttings and planted in full sun also had high relative survival (Fig. 2b).

**Table 1:** Results of binomial regression models that tested differences in survival and reproduction of 632 *Dicerandra immaculata* var. *savannarum* one year and four years post-planting due to microhabitat, collection site of the parent plant, propagation method, and planting date.

	Year 1 Survival			Year 4 Survival		
Term	*χ* ^2^	df	*P*	*χ* ^2^	df	*P*
Microhabitat	10.7	3	0.01	33.6	3	<0.001
Parent collection site	13.3	1	<0.001	9.04	1	0.003
Propagation method	9.78	1	0.002	11.4	1	0.01
Planting date	47.1	3	<0.001	0.07	3	0.79
Microhabitat x propagation	13.5	3	0.004	11.2	3	0.01

	Year 1 Reproduction			Year 4 Reproduction		
Microhabitat	2.50	3	0.47	1.59	3	0.66
Parent collection site	12.5	1	<0.001	3.22	1	0.07
Propagation method	1.44	1	0.23	7.52	1	0.006
Planting date	23.6	3	<0.001	4.88	3	0.18
Microhabitat x parent site	9.36	3	0.02	–	–	–

**Figure 3:**
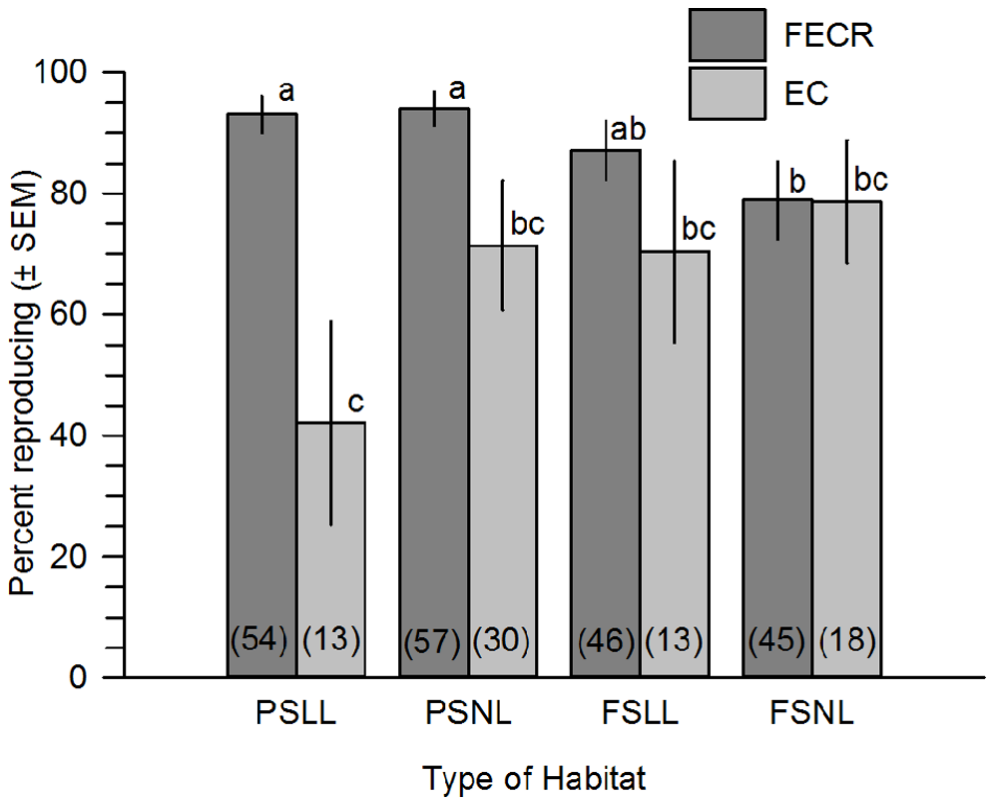
Mean percent (± SEM) of Dicerandra immaculata var. savannarum that were reproducing one year post-planting. Plants were progeny of parent plants collected from two locations and were planted in four types of microhabitat. Parent collections sites: FECR  =  Florida East Coast Railway; and EC  =  Eden Creek Lane in St. Lucie County, FL. Microhabitat types: PSLL  =  part shade and no leaf litter; PSNL  =  part shade and no leaf litter; FSLL  =  full sun and leaf litter; and FSNL  =  full sun and no leaf litter. Means with different letters are significantly different (means separation test, P<0.05).

The microhabitat and parent collection site interacted to influence reproduction of Savannas Mint one year post-planting, and planting date also was significant (Table 1). On average, more plants were reproductive one year post-planting if their parent was collected from FECR, versus EC, and planted in part shade and leaf litter, part shade and no leaf litter, or full sun and leaf litter (Fig. 3). After four years, the propagation method and parent collection site influenced reproduction, but the other terms were not significant, including microhabitat (Table 1).

**Figure 4:**
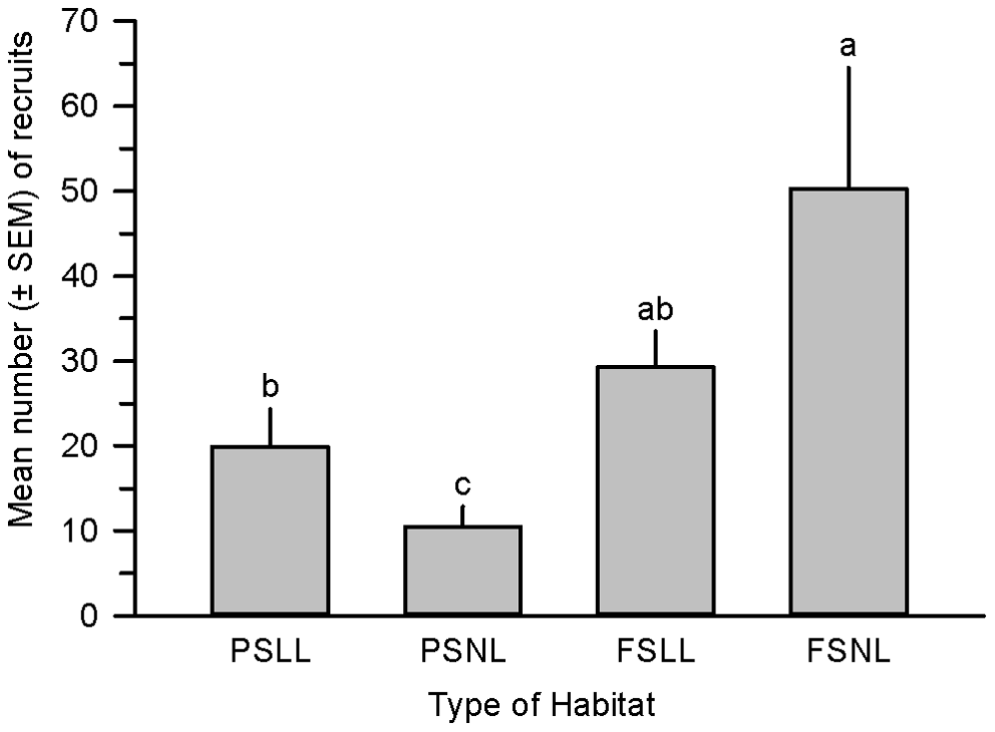
Mean number (± SEM) of Dicerandra immaculata var. savannarum plants naturally recruited to four microhabitat types. Microhabitat types: PSLL  =  part shade and no leaf litter; PSNL  =  part shade and no leaf litter; FSLL  =  full sun and leaf litter; and FSNL  =  full sun and no leaf litter. Means with different letters are significantly different (means separation test, P<0.05).

In 2011 we counted 870 new plants recruited to the population at SPSP and 276 were within the eight plots we established in the four types of microhabitat. The mean number of new recruits was influenced by the type of microhabitat (*χ* ^2^ = 64.1, df = 3, *P*<0.001) and was highest in sunny microhabitats (Fig. 4). The percent of introduced plants (i.e., parent plants of the recruits) surviving to one year post-planting and the interaction between that covariate and microhabitat also were significant (% survival, *χ* ^2^ = 3.82, df = 1, *P* = 0.051; % survival x microhabitat, *χ* ^2^ = 59.2, df = 3, *P*<0.001).

### Influence of Quench™, propagation method, parent collection site, and planting date on two-year survival and reproduction of Savannas Mint

Approximately 35% of the introduced plants treated with Quench™ survived for two years, which was not different than the 40% survival of plants that were untreated (*χ* ^2^ = 2.59, df = 1, *P* = 0.21). However, plants that were untreated were more likely to reproduce two years post-planting (88.9±3.7%) than those treated with Quench™ (78.6%±2.4%; *χ* ^2^ = 4.36, df = 1, *P* = 0.04). Plants propagated from seeds had higher survival and were more likely to reproduce two years post-planting than those propagated from clonal cuttings (Table 2). Over 20% more plants survived for two years if their parents were collected from EC than FECR, but ∼13% more plants reproduced if their parents were collected from FECR than EC (Table 2). Planting date also may influence survival and reproduction: plants had the highest survival when planted at SPSP in summer months (June and July), but reproduction was less variable among planting dates (Table 2). However, these results should be considered preliminary because we were not able to replicate the planting dates during the same months each year.

**Table 2:** Mean percent (± SEM) survival and reproduction of *Dicerandra immaculata* var. *savannarum* two years after 1,503 individual plants were planted at Savannas Preserve State Park, FL.

		Demographic Category	
Term	Variable	Mean % survival (SEM)	Mean % reproduction (SEM)
Propagation	Seed	47.0 (3.4)a	88.9 (2.7)a
	Clone	33.2 (1.8)b	79.2 (2.8)b
Parent collection site	EC	50.8 (3.1)a	77.3 (38)b
	FECR	29.9 (3.3)b	90.0 (2.7)a
Planting date	June 2006	69.9 (5.5)a	79.3 (7.7)bc
	September 2006	38.2 (3.4)b	61.0 (6.0)c
	November 2006	22.3 (4.5)c	83.8 (6.5)b
	February 2007	36.2 (4.9)bc	91.3 (4.4)ab
	October 2007	32.4 (3.8)bc	91.6 (2.6)a
	September 2008	25.8 (2.8)c	83.8 (3.7)b
	July 2009	59.3 (5.5)a	89.5 (3.7)ab

The influence of propagation method, site from which the parent was collected, and date of field planting on survival and reproduction is noted. Means followed by different letters within a term x demographic category combination are significantly different (means separation test, *P*<0.05).

### Influence of parent genotype on two-year survival of Savannas Mint

Parent genotype influenced survival of Savannas Mint (*χ* ^2^ = 149.3, df = 49, *P*<0.001). The most successful progeny of parent genotypes had a survival rate of 68.8±11.6%, which was nearly 12 times higher than the 5.8±2.3% survival rate of the least successful progeny (Table 3).

**Table 3:** Mean percent (± SEM) survival of *Dicerandra immaculata* var. *savannarum* two years after clonal progeny from 50 parent genotypes were planted.

Parent genotype no.	N	Mean % survival (SEM)	Parent genotype no.	N	Mean % survival (SEM)	Parent genotype no.	N	Mean % survival (SEM)
160	16	68.8 (11.6)a	564	15	36.4 (14.5)abcdef	558	19	22.2 (9.8)cdef
2	12	63.4 (14.5)ab	150	29	34.6 (9.3)bcdef	158	10	20.0 (12.7)cdefg
127	25	62.5 (9.9)ab	3	38	34.2 (7.7)bcdef	159	12	20.0 (12.7)cdefg
556	27	60.9 (10.2)ab	170	13	33.3 (13.6)bcdef	162	11	20.0 (12.7)cdefg
148	17	60.0 (12.7)ab	562	12	33.3 (13.6)bcdef	563	21	19.1 (8.6)defg
129	18	55.6 (11.7)ab	6	37	32.4 (7.7)bcdef	128	18	16.7 (8.8)defg
137	30	55.2 (9.2)ab	147	32	32.3 (8.4)bcdef	169	13	16.7 (10.8)efg
85	21	52.6 (11.5)abc	134	17	31.3 (11.6)bcdef	139	33	15.2 (6.2)efg
149	12	50.0 (15.8)abcd	555	27	29.6 (8.8)bcdef	143	15	14.3 (9.4)efg
157	36	46.9 (8.8)abcd	559	11	27.3 (13.4)bcdef	135	17	12.5 (8.3)efg
565	25	45.5 (10.6)abcd	133	15	26.7 (11.4)bcdef	167	18	12.5 (8.3)efg
141	13	41.7 (14.2)abcde	163	35	26.5 (7.6)cdef	7	34	12.1 (5.7)fg
161	39	41.7 (8.2)abcde	4	16	25.0 (10.8)cdef	154	27	12.0 (6.5)fg
136	23	40.9 (10.5)abcde	171	17	25.0 (10.8)cdef	138	12	10.0 (9.5)fg
554	11	40.0 (15.5)abcdef	557	12	25.0 (12.5)cdef	132	17	5.8 (5.7)fg
126	23	38.9 (11.5) abcdef	152	22	23.8 (9.3)cdef	1	103	5.8 (2.3)g
156	18	38.9 (11.5) abcdef	5	22	22.7 (8.9)cdef			

Means followed by different letters are significantly different (means separation test, *P*<0.05).

## Discussion

The amount of sunlight, leaf litter, propagation method, parent genotype, parent collection site, planting date, and absorbent granules all influenced the survival, reproduction, and/or recruitment of Savannas Mint in a large introduced population. Further controlled experiments need to be done to verify our results, but Savannas Mint apparently is adapted to open sunny habitats, warm weather, and well-drained soil, like other *Dicerandra* spp. [24], [28]. Evidence supporting this statement is our data suggesting that Quench™ had a negative influence on reproduction of Savannas Mint and plants had the highest survival when planted during hot summer months. Other *Dicerandra* spp. are adapted to well-drained soil [36] and the wild populations of Savannas Mint grew on well-drained soil [19], so it appears that Savannas Mint does better when the soil surrounding its roots are not artificially kept wet from Quench™.

Microhabitat preferences of plants are a major factor controlling distribution, abundances, and population dynamics of species [31], [37], [38], so understanding the microhabitat preference of Savannas Mint and other rare plants will help identify habitat of conservation value and identify factors that threaten survival of a species. Our results show that short and long-term survival, reproduction, and recruitment of new Savannas Mint plants to the population tends to be higher in microhabitats in full sun and no leaf litter and lower in partially shaded habitats, which is similar to *D. frutescens* Shinners and *D. christmanii* R.B. Huck & W.S. Judd [26], [36]. Whereas Savannas Mint also had high survival in microhabitats that were partially shaded and had leaf litter, recruitment was lower than in sunny habitats. Plants in the introduced population were watered after introduction and this likely improved their short-term survival and reproduction across all microhabitats, but we are unsure whether this would influence survival and reproduction over the long-term. Gaps in the canopy and low competition at ground level are likely essential for survival and recruitment of Savannas Mint, and habitat needs to be properly managed through prescribed burning and/or mechanical removal of competing plants. The overall ambient weather conditions at SPSP before and after the planting date also might interact with microhabitat to influence survival and reproduction and a population viability analysis that combines demographic and weather data would help uncover those effects.

The two sites from which parent plants were collected, FECR and EC, differentially influenced survival and reproduction of progeny. Plants survived longer if their parents were from EC, but more plants reproduced if their parents were from FECR. These differences could be due to variation in resource reserves of the parent plants between the two sites that is passed to their progeny, particularly clonal progeny. However, these differences are more likely due to underlying genetic mechanisms since differences in survival and reproduction between parent collection sites persist years after parent plants and/or progeny have been grown under similar conditions at Bok Tower Garden and SPSP. Differential survival of progeny from different parent genotypes further supports the idea that underlying genetics is an important consideration when restoring plant populations. The type of germplasm used to restore populations of Savannas Mint is also important and seedlings should be used rather than clonal cuttings, unless there are an insufficient number of seeds to produce a genetically diverse population.

In conclusion, we identified environmental, propagation, and genetic factors that will improve efforts to conserve and restore populations of Savannas Mint. We speculate that many of these factors are likely to influence allopatric congeners [24], and critically endangered gap specialists that grow in Florida scrub, and our results can be used to guide their conservation. On a broader level, our work shows that there are a myriad of environmental factors that can affect the success of conservation programs aimed at rare plant species and that genetic diversity is important to maintain because not all genotypes are equally likely to survive and reproduce. High genetic diversity in a plant population also is likely to increase the fitness of the plants in the population and increase the multitrophic diversity of associated arthropods [39], [40].
